# “Platelet/lymphocyte” and “neutrophil/lymphocyte” ratios in children with rheumatic heart involvement

**DOI:** 10.1097/MD.0000000000046201

**Published:** 2026-05-12

**Authors:** Ecem İpek Altinok, Emine Yurdakul Ertürk, Taner Kasar

**Affiliations:** a Department of Pediatrics, Faculty of Medicine, Ordu University, Ordu, Turkey; b Department of Pediatric Cardiology, Faculty of Medicine, Ordu University, Ordu, Turkey.

**Keywords:** acute rheumatic fever, neutrophil/lymphocyte ratio, platelet/lymphocyte ratio

## Abstract

The aim of this study was to determine how platelet/lymphocyte (PLR) and neutrophil/lymphocyte (NLR) ratios change in children diagnosed with Acute Rheumatic Fever (ARF) who are followed up in our clinic. Additionally, we aimed to investigate whether these values are useful in predicting the long-term development of chronic rheumatic heart disease in these patients. A total of 27 patients diagnosed with ARF with who were referred to the Pediatric Cardiology outpatient clinic between January 2019 and August 2023 were included in this study. The retrospective records of the patients were reviewed. Twenty-seven ARF patients (14 females, 13 males; mean age 10.3 ± 2.3 years) and 27 controls (12 females, 15 males; mean age 8.9 ± 3.2 years) were evaluated. Carditis was present in 85.2% of ARF cases at diagnosis, with mitral regurgitation being the most common lesion (29.6%). Leukocyte count, PLR, and NLR were significantly elevated in the ARF group compared to controls (*P* = .04, .001, .014), while lymphocyte counts were reduced (*P* = .001). Severe valve involvement was associated with lower PLR (*P* = .022), but not with NLR (*P* = .12). Although ARF incidence is declining in developed countries, it remains a significant health issue in developing regions. Consistent with global data, carditis – especially mitral regurgitation – was the predominant finding in our cohort. NLR and PLR have emerged as stable markers for cardiovascular risk, unaffected by exercise or dehydration. Our results demonstrate increased leukocyte counts, NLR, and PLR alongside decreased lymphocytes in ARF patients. Importantly, lower PLR correlated with more severe valve disease, unlike NLR. These findings highlight PLR’s potential as a prognostic biomarker, meriting further research in larger populations.

## 1. Introduction

Acute rheumatic fever (ARF) is a significant cause of acquired heart disease in children. It typically follows an upper respiratory tract infection caused by Group A beta-hemolytic streptococcus and predominantly affects children aged 5 to 15 years. The most critical factor influencing mortality and morbidity is cardiac involvement.^[1]^

While the incidence of ARF has decreased in developed countries, it remains a major health concern in developing countries. Since the 2000s, a decline in ARF incidence has been observed in developed nations, with rates ranging from 0.1 to 2 per 100,000.^[2]^ In our country, regional variations exist; a recent study reported an incidence of 8.84 cases per 100,000 in Turkey.^[3]^

The modified Jones criteria, finalized in 2015, are used for diagnosing acute rheumatic fever (ARF). Evidence of either two major criteria or one major and two minor criteria, along with proof of a recent streptococcal infection (such as throat culture, history of scarlet fever, elevated antistreptolysin O titers, and serological tests), is sufficient for diagnosis.^[4]^

The major criteria include carditis, polyarthritis, chorea, erythema marginatum, and subcutaneous nodules. Arthritis, which commonly presents as migratory, unilateral, and involving large joints, is the most frequent major criterion. Carditis is the most serious major manifestation of ARF, typically resulting in mitral insufficiency.^[5]^ The minor criteria include clinical findings such as fever and arthralgia, as well as laboratory findings such as a prolonged PR interval on ECG (electrocardiogram), elevated sedimentation rate, and increased C-reactive protein.

Studies predicting who will develop carditis in patients diagnosed with ARF and the severity of this carditis have become increasingly important. Recent research has focused on changes in neutrophil/lymphocyte ratio (NLR) and platelet/lymphocyte ratio (PLR) in rheumatological and inflammatory diseases. NLR reflects the balance between inflammation and immune regulation^[6]^ and has been shown to predict mortality and major adverse cardiac events in acute coronary syndromes and degenerative aortic stenosis.^[7,8]^ Additionally, platelets play a role in B cell-mediated immune modulation, making PLR a proposed indicator of inflammation.^[9]^

However, there are limited studies investigating changes in PLR and NLR values in ARF patients with and without carditis. The aim of our study is to determine how PLR and NLR values change in children diagnosed with ARF during follow-up and to investigate whether these parameters could be useful in predicting long-term rheumatic heart disease.

## 2. Methods

Patients diagnosed with ARF who presented to the Pediatric Cardiology outpatient clinic between January 2019 and August 2023 were included in this study. The study was approved by the Ordu University Ethics Committee with decision number 2023/236. All patients within the date range were accessed from the records, and patients with missing information were excluded. Healthy children under 18 years who presented to general pediatrics outpatient clinics for routine checkups were included as the control group. Since the study is retrospective, the total number of subjects was determined using a complete enumeration technique, and the control group was kept the same size Participants’ gender, age, presenting complaints, physical examination, laboratory data (hemogram, biochemistry, sedimentation rate, C-reactive protein), ECG, echocardiogram findings, and clinical course characteristics were examined. The echocardiogram was performed by a pediatric cardiologist, and all results, including the ECG, were evaluated by them. Cases with carditis were grouped according to the type and severity of carditis.

Data analysis was conducted using SPSS Statistics 26.0 (IBM Corp., Armonk). The normal distribution of the data was assessed using the Shapiro–Wilk test. The Mann–Whitney *U* test was used for numerical data, and chi-square analysis was employed for categorical data. In addition to reporting *P* values, effect sizes (Cohen’s *d*) and 95% confidence intervals (CIs) were calculated and interpreted to assess the magnitude and clinical relevance of differences. A significance level of *P* < .05 was considered statistically significant.

## 3. Results

A total of 27 ARF cases were included in the study, comprising 14 females and 13 males. The average age of the patients was 10.3 ± 2.3 years. The control group consisted of 27 healthy children (12 females, 15 males) with an average age of 8.9 ± 3.2 years.

Ten patients were diagnosed in 2019, 4 in 2021, 4 in 2022, and 9 in 2023. Carditis was detected in 23 patients (85.2%) at the time of diagnosis. Isolated mitral regurgitation was the most common finding (29.6%), followed by mitral and aortic regurgitation (18.5%), and isolated aortic regurgitation (14.8%). Polyarthritis was the most common symptom after carditis, occurring in 37% of cases. Demographic and clinical characteristics of the study group are shown in Table 1.

**Table 1:** Details the demographic and clinical data of patients diagnosed with acute rheumatic fever.

	Number (n)	Percent (%)
Gender		
Male	13	48.1
Female	14	51.9
Year of diagnosis		
2019	10	37
2020	0	0
2021	4	14.8
2022	4	14.8
2023	9	33.3
Valve involvement (total)	23	85.1
Isolated mitral regurgitation	8	29.6
Mitral + aortic regurgitation	5	18.5
Isolated aortic regurgitation	4	14.8
Involvement of all valves	3	11.1
Mitral + tricuspid regurgitation	3	11.1
Arthritis		
Polyarthritis	7	25.9
Monoarthritis	0	0
Arthralgia		
Polyarthralgia	3	11.1
Monoarthralgia	10	37
Sydenham chorea	3	11.1
Erythema marginatum	2	7.4
Subcutaneous nodule	0	0

The laboratory parameters of patients and healthy controls were compared and the results are presented in Table 2. Total leukocyte count was significantly higher in patients compared to controls (9464 ± 4365 vs 7302 ± 2040, *P* = .043), with a medium effect size (Cohen’s *d* = 0.60, 95% CI: 0.06–1.10). Although the neutrophil count was also higher in the patient group, this difference was not statistically significant (*P* = .842), and the effect size remained small (*d* = 0.42).

**Table 2:** Compares the laboratory characteristics of patients and controls.

	Patient (27)	Control (27)	*P* value	Cohen’s *d*	95% confidence interval
Leukocyte	9464 ± 4365	7302 ± 2040	**.043**	0.60	(0.06–1.10)
Neutrophil	6033 ± 4226	4778 ± 1289	.842	0.42	(−0.12 to 0.96)
Lymphocyte	2956 ± 2231	3757 ± 1027	**.001**	−0.44	(0.98–0.10)
Hemoglobin	12.04 ± 1.10	12.51 ± 1.21	.203	−0.45	(−0.99 to 0.09)
MCV	79.51 ± 3.01	77.80 ± 3.88	.073	0.24	(−0.29 to 0.78)
RDW	13.32 ± 1.15	13.22 ± 0.86	.959	−0.10	(−0.64 to 0.44)
Platelet	335.520 ± 105.460	304.960 ± 140.278	.149	0.35	(−0.19 to 0.89)
Platelet/lymphocyte	149.72 ± 75.93	78.03 ± 28.95	**.001**	**1.35**	(0.76–1.94)
Neutrophil/lymphocyte	2.97 ± 3.0	1.38 ± 0.61	.14	0.80	(0.25–1.36)

The values in bold indicate statistically significant results. MCV = mean corpuscular volume, RDW = red cell distribution width.

Interestingly, lymphocyte count was significantly lower in the patient group compared to controls (2956 ± 2231 vs 3757 ± 1027, *P* = .001), with a medium effect size in the opposite direction (*d* = –0.44, 95% CI: –0.98 to 0.10). Similarly, hemoglobin levels were lower in patients, albeit not statistically significant (*P* = .203), with a small-to-medium effect size (*d* = −0.45).

Mean corpuscular volume and red cell distribution width did not differ significantly between the groups (*P* = .073 and *P* = .959, respectively), and the effect sizes were minimal. Platelet counts were higher in the patient group but did not reach statistical significance (*P* = .149), with a small effect size (*d* = 0.35).

Among derived ratios, PLR showed a highly significant difference between groups (149.72 ± 75.93 vs 78.03 ± 28.95, *P* = .001), and the effect size was large (Cohen’s *d* = 1.35, 95% CI: 0.76–1.94), indicating a strong discriminatory potential. Although NLR was higher in patients (2.97 ± 3.0 vs 1.38 ± 0.61), the difference did not reach statistical significance (*P* = .14); however, the effect size was notable (*d* = 0.80, 95% CI: 0.25–1.36), suggesting a potentially meaningful clinical difference that may become significant with a larger sample size.

Table 3 presents a comparison of PLR and NLR values based on the presence of valve involvement (carditis). The mean NLR was 2.64 ± 2.8 in patients with carditis and 4.85 ± 3.89 in those without carditis. This difference was not statistically significant (*P* = .24), and the effect size was small (Cohen’s *d* = −0.38; 95% CI: −1.31 to 0.55), suggesting no meaningful association between NLR and carditis.

**Table 3:** Compares PLR and NLR parameters with valve involvement severity.

	Carditis present (n = 23)	Carditis absent (n = 4)	*P* value	Cohen’s *d*	95% confidence interval
NLR	2.64 ± 2.8	4.85 ± 3.89	0.24	−0.38	(−1.31 to 0.55)
PLR	155.87 ± 80.54	114.39 ± 21.02	0.15	0.69	(−0.25 to 1.63)

NLR = neutrophil/lymphocyte ratio, PLR = platelet/lymphocyte ratio.

In contrast, the PLR was higher in patients with carditis (155.87 ± 80.54) than in those without (114.39 ± 21.02). Although this difference did not reach statistical significance (*P* = .15), it demonstrated a moderate effect size (Cohen’s *d* = 0.69; 95% CI: −0.25 to 1.63). This finding suggests that, despite the limited sample size, PLR may be associated with the severity of valve involvement (Table 3).

**Figure 1.**
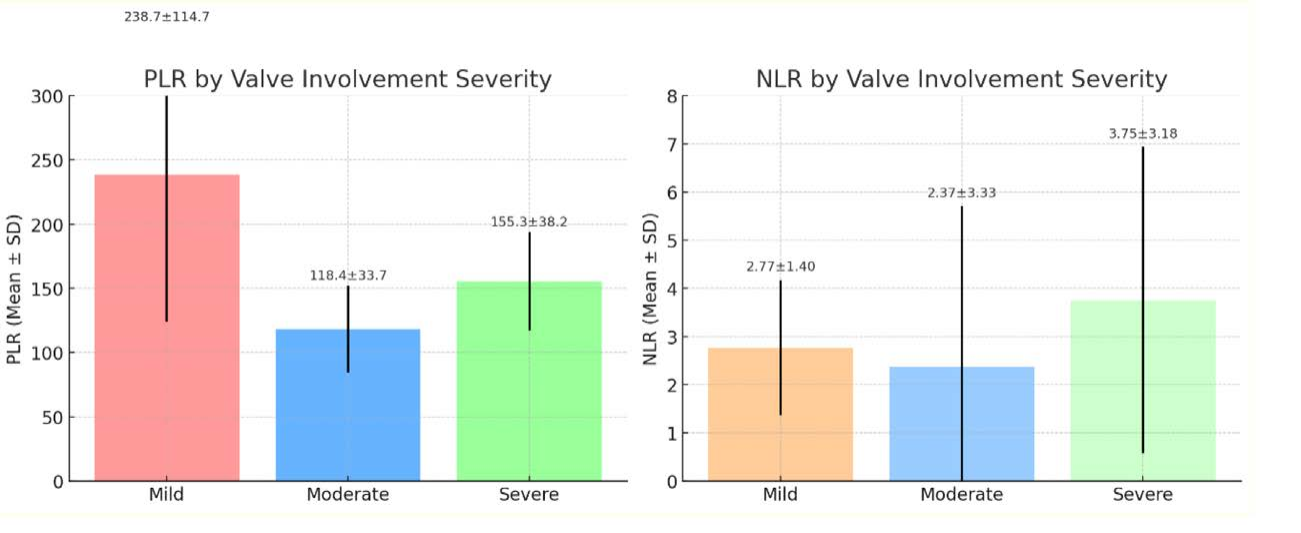
Distribution of PLR and NLR levels according to the severity of valve involvement. PLR and NLR values are shown across groups with varying severity of valve involvement. PLR and NLR are inflammatory markers used to assess disease severity. NLR = neutrophil-to-lymphocyte ratio, PLR = platelet-to-lymphocyte ratio.

The relationship between the severity of valve involvement and PLR and NLR is graphically presented in Figure 1. PLR values were found to be highest in patients with mild valve involvement, followed by a decrease in mean values as severity increased. However, the difference between the mild and moderate groups was statistically significant (*P* = .022; Cohen’s *d* = 1.35; 95% CI: 0.20–2.38). Regarding NLR, values tended to increase as the severity of valve involvement increased, but these differences were not statistically significant (*P* = .12; Cohen’s *d* = 0.16; 95% CI: −0.78 to 1.06). Nevertheless, an increasing trend in NLR levels in patients with severe valve involvement is noteworthy.

## 4. Discussion

Although the incidence of ARF has decreased in developed countries, it remains a significant health issue in developing and underdeveloped regions. The prevalence in these countries is estimated to be approximately ten times higher than in developed nations.^[2]^ Turkey is considered a moderate-to-high endemic country for ARF, with reported incidence rates ranging between 5 and 15 per 100,000 among school-aged children in different regions.^[3]^

According to the literature, carditis occurs in 50% to 75% of patients with major symptoms, polyarthritis in 75% to 80%, chorea in 10% to 15%, and erythema marginatum and subcutaneous nodules in less than 5% of patients.^[10,11]^ In our study, no subcutaneous nodules were observed, while carditis was detected in 85.2%, polyarthritis in 37%, monoarthritis in 25.9%, chorea in 11.1%, and erythema marginatum in 7.4% of cases. The frequency of major symptoms observed in our study generally aligns with the existing literature. Notably, carditis was the most common major finding.

While arthritis is often reported as the most common major finding in some studies, carditis is predominant in others. Studies conducted in our country by Karaaslan et al^[12]^ and Çağatay et al^[13]^ identified arthritis as the most frequent major finding, whereas Yavrum et al^[14]^ found isolated carditis to be the most common. These discrepancies are thought to stem from whether the study center has a cardiology department and whether ECG is routinely performed, as silent carditis can be detected through ECG. In studies conducted in our country, the rate of carditis tends to be higher in centers with a cardiology unit, while the rate of arthritis is more prevalent in studies conducted in general pediatric services.^[12–14]^

ARF remains the most common cause of acquired heart disease worldwide, with mitral valve involvement being the most frequently observed, followed by aortic valve involvement.^[15]^ In a screening study conducted by Beaton et al involving 4869 school children, mitral insufficiency was identified in 97.8% of cases with cardiac involvement.^[16]^ In our study, mitral regurgitation was detected in 19 out of 23 patients (82.6%) with valve involvement, with isolated mitral regurgitation (42%) being the most common, followed by mitral and aortic regurgitation (26%), consistent with the literature.^[17]^

Recently, there has been increasing interest in whether neutrophil and lymphocyte counts, as well as the NLR, can predict cardiovascular risk.^[18]^ NLR, considered an indicator of inflammation unaffected by exercise or dehydration, is expected to provide more accurate and objective results, leading to numerous recent studies. Some studies in our country have reported significantly higher neutrophil counts and NLR in patients with acute rheumatic carditis compared to controls, with increased NLR levels correlating positively with the severity of valve involvement.^[19,20]^ Giray and Hallioglu^[9]^ found significantly lower lymphocyte counts in both the control and carditis groups. In our study, no statistically significant difference was found in NLR values between patients with and without valve insufficiency (*P* = .24). Although NLR was higher in patients without carditis (4.85 ± 3.89) compared to those with carditis (2.64 ± 2.8), the difference was not significant and the effect size was small (Cohen’s *d* = –0.38). Conversely, a noteworthy finding was the lower PLR in patients without carditis (114.39 ± 21.02) compared to those with carditis (155.87 ± 80.54), with a moderate effect size (Cohen’s *d* = 0.69), despite the *P* value not reaching significance (*P* = .15). These findings suggest that PLR may better reflect the severity of inflammatory involvement in ARF than NLR.

Graphical analyses further demonstrated that PLR values were highest in patients with mild valve involvement, and decreased as the severity increased, with a significant difference between mild and moderate groups (*P* = .022; Cohen’s *d* = 1.35). In contrast, NLR showed an increasing trend with valve involvement severity, though this did not reach statistical significance (*P* = .12). The combined reporting of effect sizes and CIs, especially for PLR, supports a clinically relevant association. These observations indicate that PLR might serve as a more sensitive marker in early inflammatory stages, whereas NLR may increase as the disease progresses.

These findings are consistent with those reported by Giray and Hallioglu, who showed that PLR and NLR are elevated in ARF patients and can serve as markers of inflammation and disease activity.^[9]^ They emphasized that PLR may be particularly sensitive to early inflammatory changes. Similarly, Aryani et al found a strong positive correlation between NLR and carditis severity, whereas PLR showed a weaker but still relevant association.^[21]^ Their results suggested that NLR may more closely reflect the progression and severity of valve involvement, while PLR might better indicate early inflammatory response.

One of the main limitations of our study is the relatively small sample size of 27 patients. Due to the retrospective single-center design, this limited number reduces the statistical power and generalizability of our findings. Additionally, public health measures such as social distancing and mask-wearing during the coronavirus disease 2019 pandemic between 2020 and 2022 reduced hospital admissions and, consequently, ARF diagnoses. Notably, no cases were diagnosed in 2020, highlighting the pandemic’s impact. The distribution of cases over the study period was 10 (37%) in 2019, 4 (14.8%) in both 2021 and 2022, and 9 (33.4%) in 2023. Considering Turkey’s endemic status for ARF, this limited sample underscores the need for larger multicenter and prospective studies to improve the generalizability of results.

Moreover, as a retrospective study, this work is inherently prone to selection and information biases. To mitigate these, we included all eligible patients within the study period except for those with missing data, and data extraction was performed systematically from electronic medical records. Nonetheless, prospective studies are needed to confirm these results and strengthen the evidence base.

The limited sample size prevented robust sex-stratified analyses. Such subgroup analyses would be statistically underpowered and prone to type II error, limiting the reliability of gender-based conclusions. We acknowledge that sex-specific immune and inflammatory responses may influence PLR and NLR values and highlight this as an important consideration for future research.

In conclusion, both PLR and NLR show promise as biomarkers in acute rheumatic fever, with PLR potentially serving as a more sensitive indicator in the early stages and NLR correlating with increasing disease severity. Nevertheless, the limited sample size of our study and previous research underscores the need for larger prospective studies to better define these associations and their clinical relevance.

## Acknowledgments

We would like to thank Associate Professor Dr Ceren Varer Akpinar for her contributions to the statistical section. This Acknowledgments is made with her permission.

## Author contributions

**Conceptualization**: Ecem Ipek Altinok, Emine Yurdakul Ertürk.

**Data curation**: Ecem Ipek Altinok.

**Investigation**: Emine Yurdakul Ertürk.

**Methodology**: Ecem Ipek Altinok.

**Resources**: Ecem Ipek Altinok.

**Supervision**: Taner Kasar.

**Validation**: Taner Kasar.

**Writing – original draft**: Ecem Ipek Altinok.

**Writing – review & editing**: Ecem Ipek Altinok.
